# A Comparative Analysis of Super-Enhancers and Broad H3K4me3 Domains in Pig, Human, and Mouse Tissues

**DOI:** 10.3389/fgene.2021.701049

**Published:** 2021-11-24

**Authors:** Yanling Peng, Huifang Kang, Jing Luo, Yubo Zhang

**Affiliations:** Animal Functional Genomics Group, Shenzhen Branch, Guangdong Laboratory for Lingnan Modern Agriculture, Genome Analysis Laboratory of the Ministry of Agriculture, Agricultural Genomics Institute at Shenzhen, Chinese Academy of Agricultural Sciences, Shenzhen, China

**Keywords:** super-enhancers, broad H3K4me3 domains, functional conservation, tissue-specific, pig

## Abstract

Super-enhancers (SEs) and broad H3K4me3 domains (BDs) are crucial regulators in the control of tissue identity in human and mouse. However, their features in pig remain largely unknown. In this study, by integrative computational analyses of epigenomic and transcriptomic data, we have characterized SEs and BDs in six pig tissues and analyzed their conservation in comparison with human and mouse tissues. Similar to human and mouse, pig SEs and BDs display higher tissue specificity than their typical counterparts. Genes proximal to SEs and BDs are associated with tissue identity in most tissues. About 55–182 SEs (5–17% in total) and 99–309 BDs (8–16% in total) across pig tissues are considered as functionally conserved elements because they have orthologous SEs and BDs in human and mouse. However, these elements do not necessarily exhibit sequence conservation. The functionally conserved SEs are correlated to tissue identity in majority of pig tissues, while those conserved BDs are linked to tissue identity in a few tissues. Our study provides resources for future gene regulatory studies in pig. It highlights that SEs are more effective in defining tissue identity than BDs, which is contrasting to a previous study. It also provides novel insights on understanding the sequence features of functionally conserved elements.

## Introduction

**FIGURE 1:**
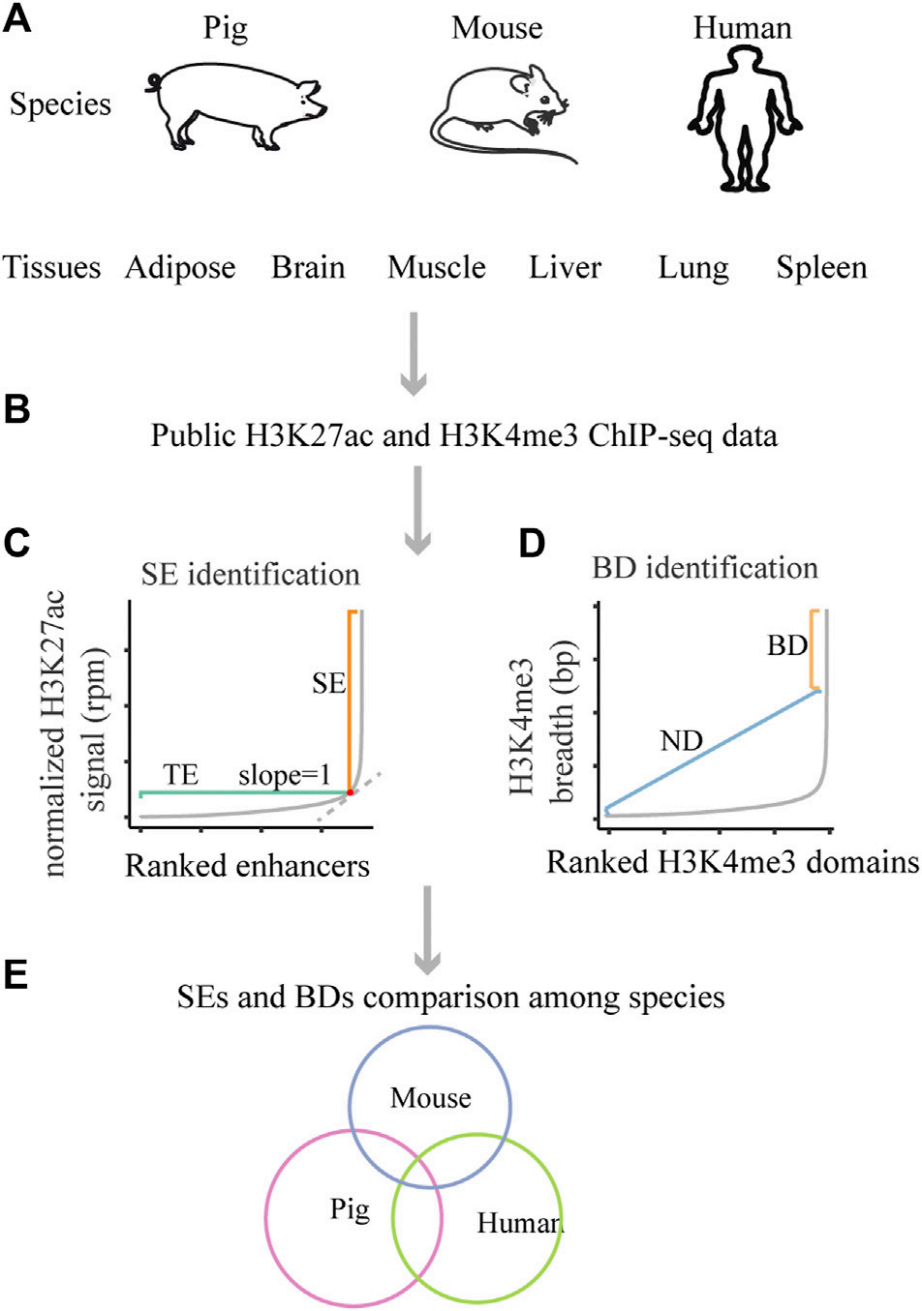
Schematic of the design of this study. **(A)** The studied species and tissues. **(B)** The used public data. **(C)** The schematic for SEs identification. **(D)** The schematic for BDs identification. **(E)** The schematic for SEs and BDs comparison among pig, human and mouse.

As clusters of enhancers, super-enhancers (SEs) are densely occupied by the master transcription regulators and histone modifications, which act as switches to determine cell/tissue identity (Hnisz et al., 2013; Whyte et al., 2013; Pott and Lieb, 2015; Peng and Zhang, 2018). Based on chromatin immunoprecipitation and high-throughput sequencing (ChIP-seq), this term was first described as clusters of enhancers with high levels of five master transcription factors (Oct4, Sox2, Nanog, Klf4, and Esrrb) and the mediators in mouse embryonic stem cells (mESCs) (Whyte et al., 2013). Later, this concept was used to describe clusters of enhancers that are densely occupied by high levels of H3K4me1, H3K27ac, p300, or master transcription factors in various mouse and human tissues (Hnisz et al., 2013; Whyte et al., 2013). Compared with other chromatin marks, H3K27ac was considered as a better mark in distinguishing SEs from typical enhancers (TEs) (Hnisz et al., 2013). H3K27ac ChIP-seq data were widely used for identifying SEs in subsequent studies (Khan and Zhang, 2016; Wei et al., 2016; Perez-Rico et al., 2017), and the algorithm for identifying SEs could be summarized as four steps (Pott and Lieb, 2015; Peng and Zhang, 2018). First, enhancers were identified based on H3K27ac enriched peaks. Second, enhancers within 12.5 kb of each other were merged. Third, the merged and individual enhancers were ranked by the background-normalized H3K27ac signal (reads per million mapped reads, rpm). Fourth, the distribution of ranked H3K27ac signal was plotted (Figure 1C), the cutoff was the point at which the slope of the plot was 1; the enhancer regions above the cutoff were designated as SEs, and the remaining were TEs. SEs usually differ from TEs in genome size, transcription factor density and content, as well as transcription ability (Hnisz et al., 2013; Whyte et al., 2013).

At the same time, broad H3K4me3 domains (BDs) are considered as another important regulatory element and could also determine cell/tissue identity (Benayoun et al., 2014). A pioneer study defined the top 5% broadest H3K4me3 domains as BDs (Benayoun et al., 2014) and we used that definition for this work (Figure 1D). Later, scientists assigned BDs as H3K4me3 domains wider than 4 kb (Chen et al., 2015). Also, scientist identified BDs based on inflection points of the H3K4me3 peak length versus gene rank (Belhocine et al., 2021). The application of SEs and BDs greatly promote the dissection of mechanism of tissue-specific processes in human and mouse (Pott and Lieb, 2015; Hay et al., 2016; Park et al., 2020).

Comparative analyses of SEs and BDs among species are important for understanding their conservation (Dincer et al., 2015; Perez-Rico et al., 2017; Luan et al., 2019), which provide the basis for dissecting the regulatory mechanisms from the evolutionary view (Snetkova et al., 2021). Based on the comparisons of SEs in pluripotent state, brain, heart, intestine, and testis across zebrafish, human, and mouse, it has been found that SEs are involved in tissue identity–related processes among these species (Perez-Rico et al., 2017). Interestingly, gene ontology (GO) annotations of pluripotent state SEs show enriched terms related to early development and pluripotency among zebrafish, human, and mouse (Perez-Rico et al., 2017). Furthermore, it has also reported that SEs that maintain orthologous gene associations among species are more conserved than those non-associated SEs (Perez-Rico et al., 2017). For the comparisons of BDs among human postmortem, non-human primate, and mouse, GO annotations of BDs in prefrontal brain cortex show enriched terms related to neuronal connectivity, development, and synaptic plasticity and learning, while BDs in blood cells are enriched GO terms for immune system–related categories (Dincer et al., 2015). However, until now, the regulatory features and conservation of SEs and BDs in pig are largely unknown.

The pig is an important species in agriculture industry and a good biomedical model, as it exhibits large similarity to humans in many aspects, such as anatomy, physiology, and genomics (Kokta et al., 2004; Meurens et al., 2012; Stower, 2018). So far, ChIP-seq and RNA-seq data in a few pig tissues are available with the initiation of Functional Annotation of Animal Genomes project (FAANG) (Andersson et al., 2015; Kern et al., 2021). Other than that, ChIP-seq and RNA-seq data in vast human and mouse tissues could be recovered with the launch of Encyclopedia of DNA Elements (ENCODE) (Moore et al., 2020) and Roadmap Epigenome Project (Kundaje et al., 2015). These projects have provided abundant resources for the identification and comparative analysis of SEs and BDs among pig, human, and mouse. The design of this study is shown in Figure 1. This study highlights the regulatory features of SEs and BDs in pig and their conservation with human and mouse.

## Materials and Methods

### Available Public Data

We downloaded H3K27ac and H3K4me3 ChIP-seq and RNA-seq data for six pig tissues (Figures 1A,B), *i.e*., adipose, skeletal muscle, brain cortex, liver, lung, and spleen, publicly. For human and mouse, we downloaded the available H3K27ac and H3K4me3 ChIP-seq and RNA-seq data for tissues such as adipose, brain (forebrain or brain cortex), muscle, liver, lung, and spleen (Figures 1A,B). The individual accession IDs are listed in Supplementary Table S1.

### ChIP-Seq Data Processing

For pig ChIP-seq data, the fastq raw files were trimmed by using Trim_galore (https://www.bioinformatics.babraham.ac.uk/projects/trim_galore/) with default parameters in single-end mode. Thus, reads with low quality (Q < 20) and shorter than 20 bp were removed. Then, high-quality reads were aligned to the pig reference genome (susScr11) using bowtie2 (Langmead and Salzberg, 2012) with default parameters. Reads with low-mapping quality scores (MAPQ <30), unmapped, and duplicated were filtered (-F 1796) using samtools (Li et al., 2009). The filtered reads were used for downstream analyses. For human and mouse ChIP-seq data, uniquely mapped reads were directly recovered from ENCODE, which were aligned to the human and mouse genomes (hg38 and mm10), respectively. H3K27ac and H3K4me3 peaks were identified using macs2 (Zhang et al., 2008) with parameters “--keep-dup = auto”, while “-g” was set to “2.7e9”, “hs”, and “mm” for pig, human, and mouse tissues, respectively. For tissues with two replicates, their peaks overlapped at least 50% reciprocally and were used for downstream analyses using bedtools (Quinlan and Hall, 2010) with parameters “intersect -f 0.5 -r”. For tissues without replicates, their top 20,000 highest signals regions were used for downstream analyses. For analyses on H3K4me3 peak signal intensity, similar to Benayoun et al. (2014), pile-up of ChIP-seq reads mapped within the significant macs2 peak was computed as the coverage in the specified intervals using function “coverage” of bedtools with default settings and normalized to the input signal in the same interval. Then, H3K4me3 peak signal was normalized to peak breadth to obtain tags per base pair of peak.

### RNA-Seq Data Processing

For pig RNA-seq data files, adapters were trimmed similar to ChIP-seq data but with paired-end mode (https://www.bioinformatics.babraham.ac.uk/projects/trim_galore/). Similar to Kern et al. (2021), reads were aligned to pig genome using STAR (Dobin et al., 2013) (--outFilterMultimapNmax 20 --alignSJoverhangMin 8 --alignSJDBoverhangMin 1 --outFilterMismatchNmax 999 --alignIntronMin 20). Meanwhile, reads with low-mapping quality scores (MAPQ <30) were filtered using samtools (Li et al., 2009). For human and mouse RNA-seq, reads that uniquely aligned to hg38 and mm10 genome were directly recovered from ENCODE. Gene counts were calculated based on bedtools (Quinlan and Hall, 2010) with function “multicov”. The gtf annotation files of pig (Suscra 11), human (hg38), and mouse (mm10) (Ensemble genes annotation) were downloaded from UCSC table browser (http://www.genome.ucsc.edu/cgi-bin/hgTables). Gene expressions were normalized to reads per million mapped reads per kilobase (rpkm) with house script.

### Identification of Super-Enhancers and Broad H3K4me3 Domains

SEs were identified based on H3K27ac enriched peaks using ROSE (https://bitbucket.org/young_computation/rose) with default parameters. Broad H3K4me3 domains were defined as the top 5% broadest H3K4me3 enriched peaks (Benayoun et al., 2014). A SE or BD was assigned to the gene with the closest transcription start site using R package ChIPseeker (Yu et al., 2015). GO analyses were performed using R package ClusterProfiler (Yu et al., 2012). GO terms with *p* values <0.001 were considered significantly enriched. The calculation of tissue specificity of SEs and BDs and their typical counterparts was based on the function “mergePeaks” in Homer (Heinz et al., 2010), and parameter “-d” was set to “2000”, “4,000”, and “8,000”, respectively.

### Comparison of Orthologous Super-Enhancers and Broad H3K4me3 Domains

For the orthologous SE and BD comparisons (Figure 1E), SEs and BDs for different tissues in pig (sucScr11) or mouse (mm10) were converted into human (hg38) coordinates by using UCSC LiftOver tools (https://genome.ucsc.edu/cgi-bin/hgLiftOver), the minimum match of which was set to 0.2. The identification of functionally conserved SEs and BDs was based on the function “mergePeaks” in Homer (Heinz et al., 2010) using default parameters. Sequence conservation scores were calculated based on the vertebrate conservation phastCons tracks (phastCons100way) from UCSC table browser.

## Results

### H3K27ac Marks Super-Enhancers and H3K4me3 Determines Broad H3K4me3 Domains in Pig

**FIGURE 2:**
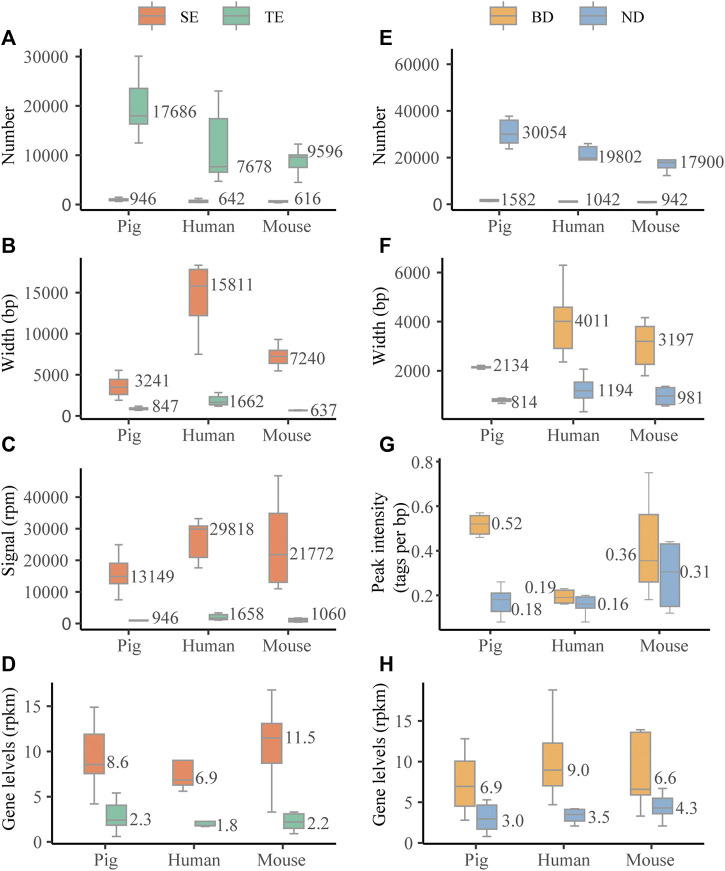
Characterization of SEs and BDs. **(A–D)** The distribution of numbers, length (bp), signal levels (reads per million mapped reads, rpm), and associated gene levels (reads per million mapped reads per kilobase, rpkm) based on the median values of six tissues across three species. **(E, F)** The distribution of numbers, length (bp), peak signal intensity (tags per bp), and associated gene levels (rpkm) based on the median values of six tissues across three species.

We first investigated the characteristic features of SEs in six pig tissues, *i.e*., adipose, skeletal muscle, brain cortex (brain), liver, lung, and spleen. For the comparisons, we also assessed the characteristic features of SEs in six human and mouse tissues, *i.e.*, adipose, skeletal muscle, brain, liver, lung, and spleen. We identified a median of 946, 642, and 616 SEs for pig, human, and mouse, respectively (Figure 2A). Pig SEs (in median, 3,241 bp) were longer than TEs (in median, 847 bp) in each tissue (Wilcoxon rank-sum test, *p* < 0.001) (Figure 2B and Supplementary Table S2). The H3K27ac signal levels of pig SEs (in median, 13,149 rpm) were higher than TEs (in median, 946 rpm) in each tissue (Wilcoxon rank-sum test, *p* < 0.001) (Figure 2C and Supplementary Table S2). Furthermore, expression levels of pig SE-associated genes (in median, 8.6 rpkm) were higher than TE-associated genes (in median, 2.4 rpkm) (Wilcoxon rank-sum test, *p* < 0.001) (Figure 2D and Supplementary Table S2). The results suggest that the characteristic features of SEs in pig are similar to human and mouse in our study and previous studies (Hnisz et al., 2013; Whyte et al., 2013).

We then examined the characteristic features of BDs in six pig tissues and compared them with six human and mouse tissues. We identified a median of 1,582, 1,042, and 942 BDs for pig, human, and mouse (Figure 2E). Similar to human and mouse, pig BDs (in median, 2,134 bp) were longer than narrow peaks (NDs) (in median, 814 bp) in each tissue (Wilcoxon rank-sum test, *p* < 0.001) (Figure 2F and Supplementary Table S3). In addition, the median peak signal intensity (tags per bp) of BDs was higher than NDs in pig, human, and mouse tissues (Figure 2G and Supplementary Table S3). Peak signal intensity was measured as the normalized number of mapped ChIP reads per base pair for each peak determined by macs2 (Benayoun et al., 2014). Values were normalized to the sequencing depth of the ChIP sample and peak breadth. Meanwhile, the levels of BD-associated genes (in median, 6.9 rpkm) were higher than NDs (in median, 3.0 rpkm) (Wilcoxon rank-sum test, *p* < 0.001) (Figure 2H and Supplementary Table S3). This indicates that there are no consistent relationships between H3K4me3 signal levels and width.

### Super-Enhancers and Broad H3K4me3 Domains Display Higher Tissue Specificity than their Typical Counterparts in Pig

**FIGURE 3:**
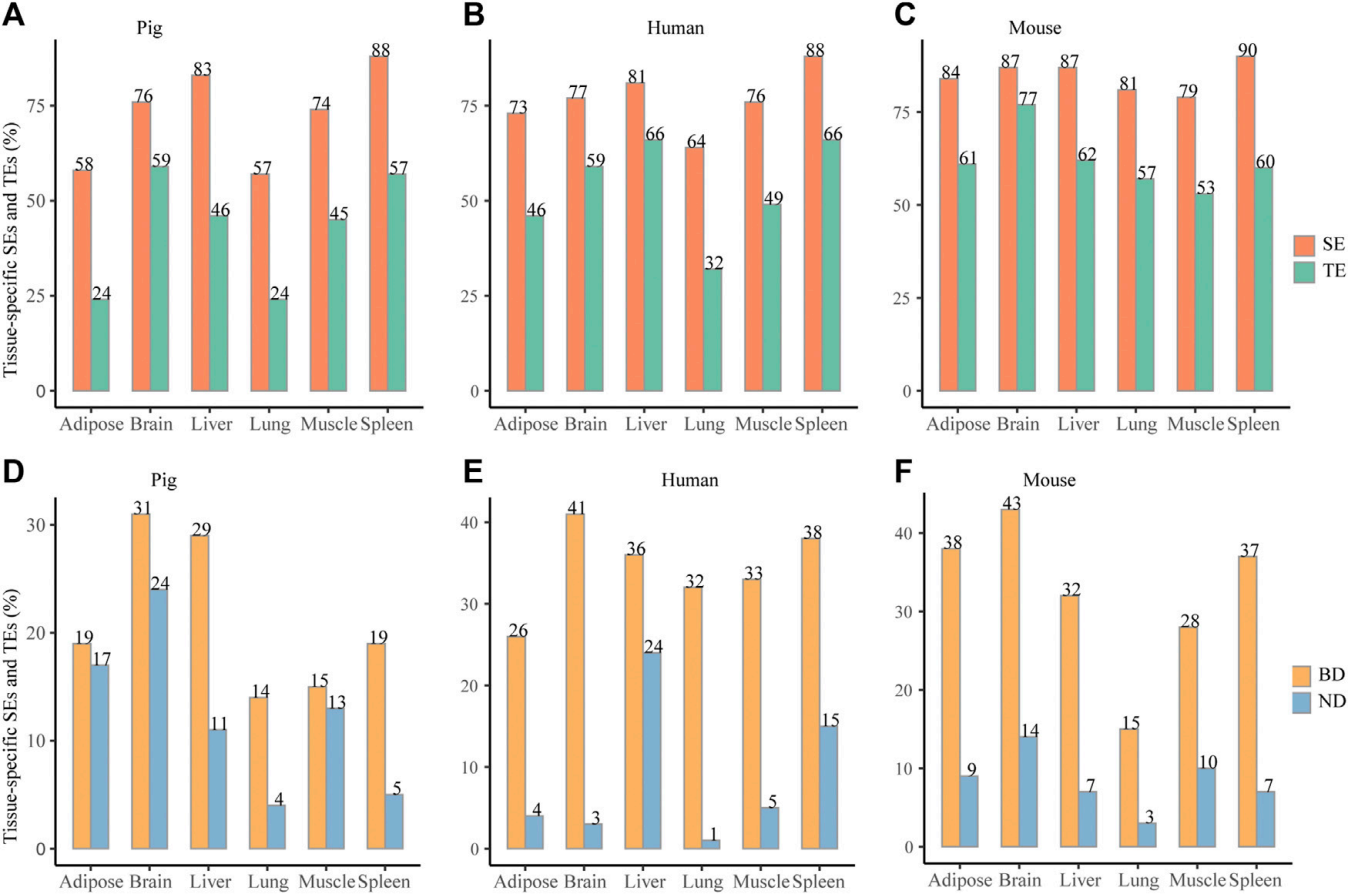
Tissue specificity of SEs and BDs. **(A–C)** Distribution of the tissue-specific SEs and TEs for pig, human, and mouse, respectively. **(D–F)** Distribution of the tissue-specific BDs and NDs for pig, human, and mouse, respectively. *** represents *p* value <0.001 based on the Wilcoxon rank-sum test.

To compare the tissue specificity between SEs and TEs, all elements were unified to the size of 2 kb according to peak median location. Across six pig tissues, about 57–83% SEs and about 32–66% TEs were tissue specific (paired Wilcoxon test, *p* < 0.05) (Figure 3A). Consistently, across six human tissues, about 64–88% SEs and about 32–66% TEs were tissue specific (Figure 3B) (paired Wilcoxon test, *p* < 0.05). Across six mouse tissues, about 79–90% SEs and about 53–77% TEs were tissue specific (paired Wilcoxon test, *p* < 0.05) (Figure 3C). When elements were unified to 4 and 8 kb, it also showed higher tissue specificity in SEs than in TEs (paired Wilcoxon test, *p* < 0.05) (Supplementary Table S4). This indicates that SEs exhibit higher tissue specificity than TEs in pig, human, and mouse.

When BDs and NDs were unified to 2 kb, about 14–31% BDs and about 4–24% NDs were tissue specific (paired Wilcoxon test, *p* < 0.05) (Figure 3D). At the same time, about 26–41% BDs and 1–24% NDs were tissue specific for six human tissues (paired Wilcoxon test, *p* < 0.05) (Figure 3E). In addition, about 15–43% BDs and 3–14% were tissue specific for six mouse tissues (paired Wilcoxon test, *p* < 0.05) (Figure 3F). When BDs and NDs were unified to 4 and 8 kb, the frequency of the tissue-specific BDs were also higher than NDs (Supplementary Table S4) (the paired Wilcoxon test, *p* < 0.05). This suggests that tissue specificity of BDs is higher than NDs in three species.

### Super-Enhancers and Broad H3K4me3 Domains are Related to Tissue Identity in Majority of Pig Tissues

**FIGURE 4:**
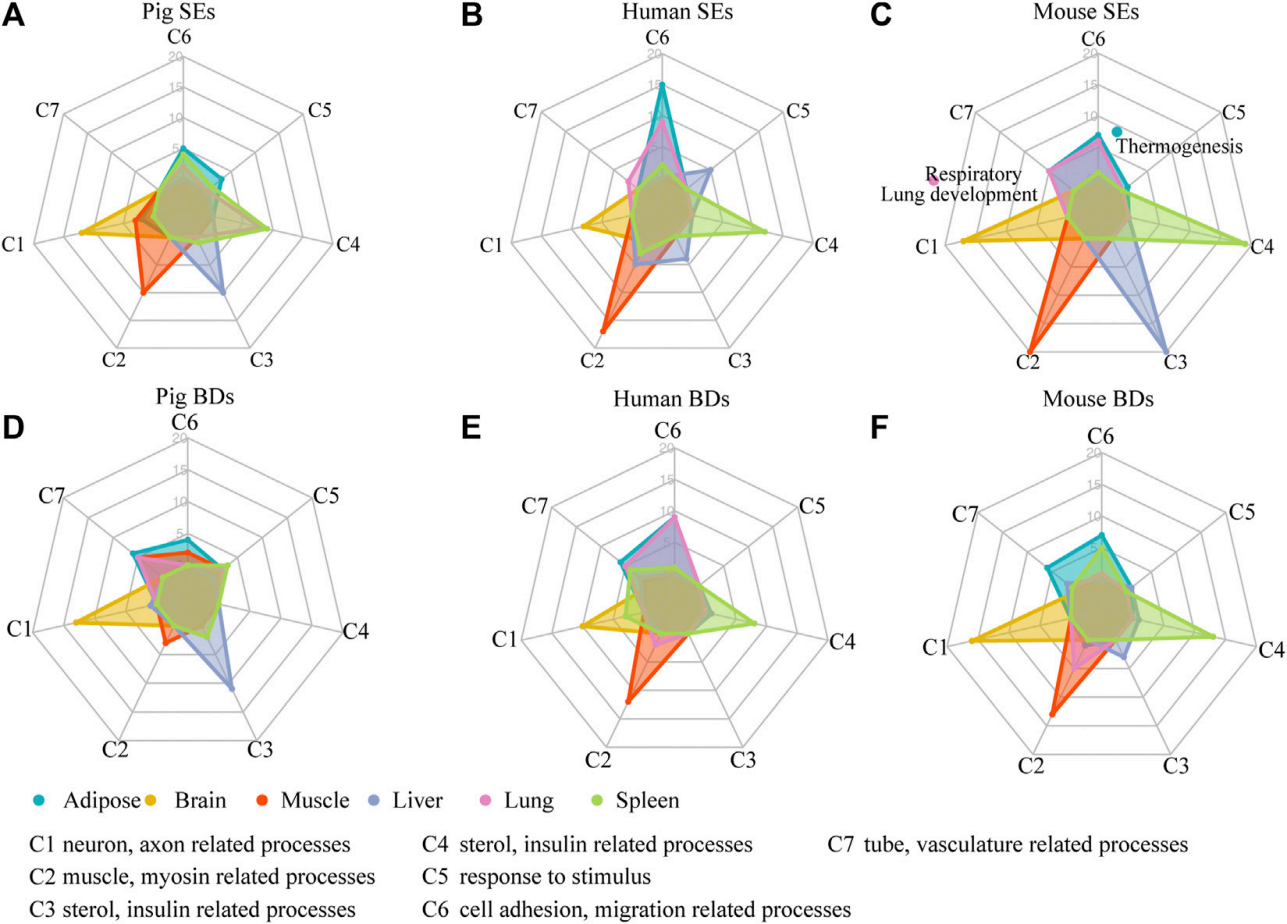
Functional analysis of SEs and BDs. **(A–C)** Radar plots showing the number of enriched GO terms of SE-associated genes in seven categories for six pig, human, and mouse tissues, respectively. **(D–F)** Radar plots showing the number of enriched GO terms of BD-associated genes in seven categories for six pig, human, and mouse tissues, respectively. Note: The top 20 most significantly enriched GO terms are selected, and the details of the GO enriched terms and categories are provided in Supplementary Table S5.

To assess whether SEs and BDs were associated with tissue identity, we classified the top 20 most significant enriched GO terms of their associated genes into seven categories (C1–C7), respectively. Each category represented tissue-specific function (Figure 4A and Supplementary Tables S4, S5). In brief, C1 corresponds to neuron, axon, and synapse-related processes. C2 contained muscle, actin, and myosin-related processes. C3 consisted of sterol metabolic, organic anion transport, insulin-related processes. C4 was composed of immune-related processes. C5 represented processes such as response to stimulus. C6 contained processes such as cell migration, adhesion, and assembly. C7 contained tube and vasculature-related processes. The details of enriched terms in each category are provided in Supplementary Table S5. For pig cerebellum, brain cortex, and hypothalamus, they showed similar enriched terms such as neuron, nervous, axon, and synapse-related processes (Supplementary Table S5). For the comparisons, brain cortex was selected at random for the SE and BD comparison with human and mouse brains.

We then used these categories (C1–C7) to explore whether SEs were involved in tissue identity–related processes in each tissue (Figures 4A–C and Supplementary Table S5). Except for pig lung, in five out of six tissues among three species, GO annotations showed SE-associated genes were enriched terms relating to tissue identity. For example, in brain, they were related to neuron-related processes (C1); in muscle, they were associated with muscle and myosin-related processes (C2); in liver, they were correlated with small molecule catabolic processes (C3); in spleen, they were correlated with immune-related processes (C4); and in adipose, they were enriched in cell mobility and cell migration–related processes (C6) (Figure 4A). In pig lung, the top 20 terms were largely distributed in immune-related processes (C4), while in human and mouse lung, they were mainly distributed in cell mobility, cell migration (C6), and tube and vasculature-related processes (C7). Moreover, in mouse lung, SE-associated genes were related to respiratory system/tube development and lung development. Our results indicate that SEs are preferable to define tissue identity in most tissues of three species except for pig lung.

At the same time, we investigated whether BDs were related to tissue identity in each tissue based on the seven categories (C1–C7) (Figures 4D–F). BDs were well coupled with tissue identity in three out of six pig tissues, *i.e.*, brain, liver, and adipose. For human BDs, they had connection with tissue identity in four human tissues, *i.e.*, brain, muscle, spleen, and adipose. For mouse BDs, they were also capable of defining four tissues, *i.e.*, brain, muscle, spleen, and adipose. This demonstrates BDs have the ability to define tissue identity in majority of tissues in pig, human, and mouse, but show a weaker determination ability than SEs.

### Functionally Conserved Super-Enhancers and Broad H3K4me3 Domains do not Necessarily Exhibit Sequence Conservation

**FIGURE 5:**
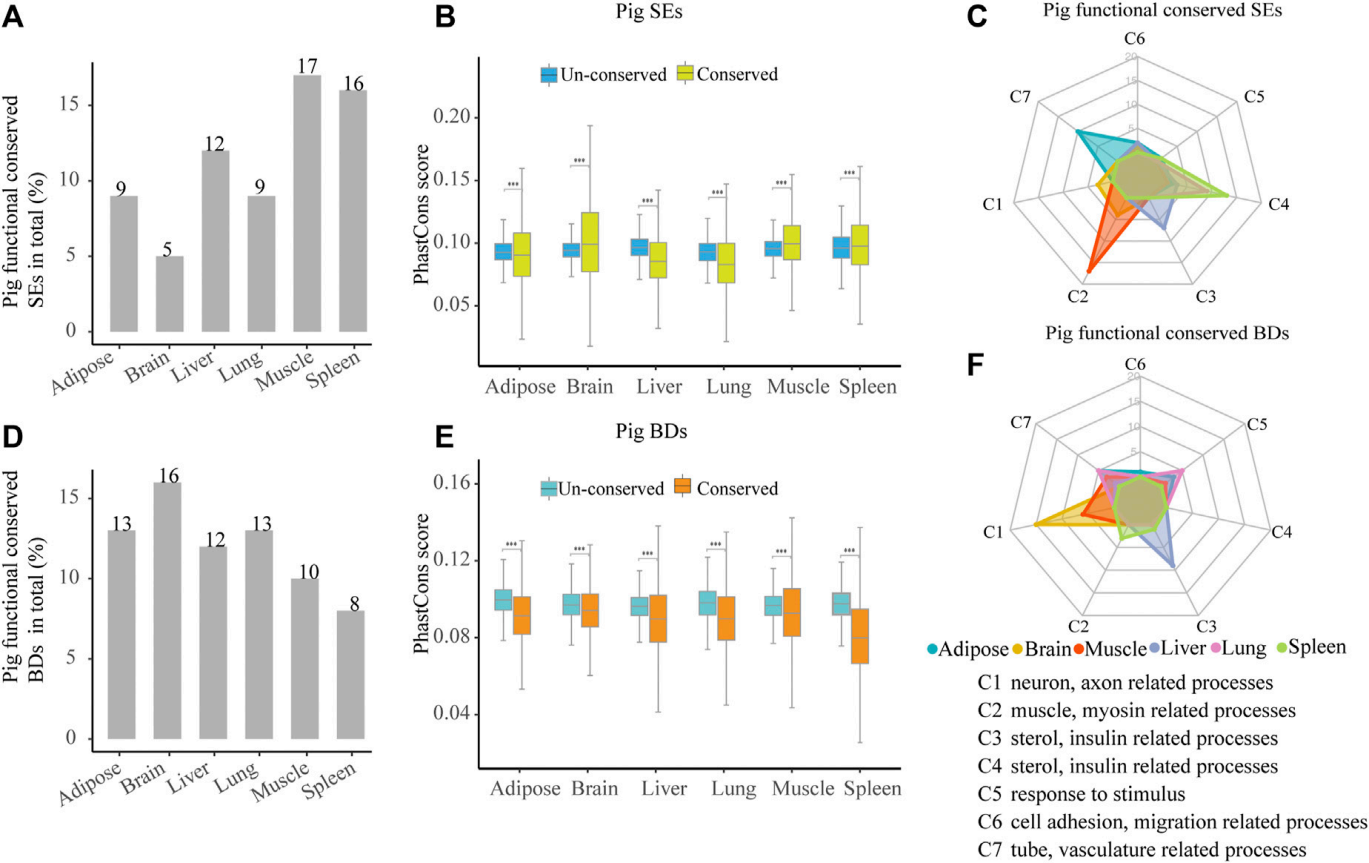
Characterization of pig functional conserved SEs and BDs. **(A)** Distribution of pig functionally conserved SEs. **(B)** Comparisons of phastCons scores between pig functionally conserved and un-conserved SEs in six tissues. **(C)** Radar plot showing the number of enriched GO terms of pig functionally conserved SE-associated genes in seven categories for six tissues. **(D)** Distribution of pig functionally conserved BDs. **(E)** Comparisons of phastCons scores between pig functionally conserved and un-conserved BDs in six tissues. **(F)** Radar plot showing the number of enriched GO terms of pig functionally conserved BD-associated genes in seven categories for six tissues. Note: The top 20 most significantly enriched GO terms are selected, and the details of the GO enriched terms and categories are provided in Supplementary Table S5.

To investigate the functional conservation of SEs among species, we analyzed the distribution of orthologous SEs in six tissues among pig, human, and mouse. Across six tissues, about 5–12% (55–182) pig functionally conserved SEs were identified because they had orthologous SEs in human and mouse (Figure 5A, Supplementary Figure S1 and Supplementary Table S6). Then, we estimated the sequence conservation of these functionally conserved SEs (Figure 5B). In pig brain, muscle, and spleen, phastCons scores in functionally conserved SEs were significantly higher than un-conserved ones, while the contrast pattern was found in pig adipose, liver, and lung (Wilcoxon rank-sum test, *p* < 0.001) (Figure 5B). Thus, this implied that functionally conserved SEs were not sequence conserved. Later, we explored the possible biological roles of the pig functionally conserved SEs. We also classified the top 20 most significant terms into seven categories (C1–C7) for six tissues. In four out of six pig tissues, including adipose, muscle, liver, and spleen, these SEs are able to control tissue identity–related genes (Figure 5C and Supplementary Table S5). The result indicates that pig functionally conserved SEs are able to define tissue identity in majority of tissues.

Likewise, to explore the functional conservation of BDs, we analyzed the distribution of BD-associated orthologous genes in six tissues among pig, human, and mouse. About 8–16% (99–309) of pig functionally conserved BDs were determined as they kept orthologous BDs in human and mouse (Figure 5D, Supplementary Figure S2, and Supplementary Table S6). Then, we estimated the sequence conservation of these functionally conserved BDs (Figure 5E). In all tissues, pig functionally conserved BDs showed lower phastCons scores than their un-conserved BDs (Wilcoxon rank-sum test, *p* < 0.001) (Figure 5E), while in brain and muscle, the patterns were reversed. This suggested that functionally conserved BDs were not sequence conserved in all detected pig tissues, which was similar to pig functionally conserved SEs. Later, we investigated the functional features of the pig functionally conserved BDs by using the seven categories (C1–C7) to summarize the top 20 most significant terms in each tissue. In contrast to SEs, conserved BDs were well relevant to tissue identity in brain and liver (Figure 5F and Supplementary Table S5). This may indicate that the ability of functionally conserved BDs in defining tissue identity is weaker than SEs.

## Discussion

SE and BD identification are critical for dissecting tissue-specific processes in human and mouse. In this study, we have characterized SEs and BDs for six pig tissues. Similar to human and mouse, pig SEs and BDs are more tissue specific than TEs and NDs, respectively. In addition, genes adjacent to pig SEs and BDs are also able to determine tissue identity in most tissues (Hnisz et al., 2013; Whyte et al., 2013; Benayoun et al., 2014). At the same time, we have detected the median peak intensity for pig BDs that is higher than for NDs, which is similar to that found in human and mouse. Our study has extended the usage of SEs and BDs and deepened the understanding of their regulatory features across mammals. In addition, it also provides tissue-specific regulatory element candidates for future functional tests in pig.

Sequence conservation between species is widely used to identify functional elements within genomes (Prabhakar et al., 2006; Consortium et al., 2007; Cooper and Brown, 2008). A previous study has revealed that highly conserved enhancer sequences can regulate fundamental processes, such as embryonic development (Prabhakar et al., 2006). In contrast, our study has found that a small portion of pig SEs (5–17% in total, 55–182) exhibit orthologous SEs in human and mouse. Especially, in four out of six tissues, including adipose, muscle, liver, and spleen, these functionally conserved SEs are related to tissue-specific processes. Based on phastCons scores, it indicates that these functionally conserved SEs do not necessarily exhibit higher scores than non-conserved ones. Consistently, a recent study suggests that the function of enhancer activities does not necessarily require the perfect sequence conservation (Snetkova et al., 2021). In fact, a previous study has also indicated that rarely conserved enhancers have overlapping functions even in phylogenetically distant species (Clarke et al., 2012; Villar et al., 2015). On the contrary, the comparisons of SEs among zebrafish, human, and mouse tissues reveal that SEs maintaining orthologous gene associations are more sequence conserved than the non-associated ones (Perez-Rico et al., 2017). The controversy may result from the comparisons among different species, which represent different evolutionary distances, while the sequences of pig functionally conserved BDs are less conserved than their non-conserved BDs in the six detected tissues, which may suggest that there is selection against sequence being conserved for pig functionally conserved BDs. Therefore, our study deepens the understanding of regulatory elements from the evolutionary view.

Our study reveals that pig functionally conserved SEs are well related to tissue identity in four out of six tissues, while conserved BDs are linked to tissue identity in two out of six tissues. Meanwhile, it reflects SEs are more efficient in determining tissue identity than BDs in pig, human, and mouse tissues. However, a pioneer study (Benayoun et al., 2014) has suggested that BDs and SEs are similarly effective at predicting validated stem cell regulators in mESCs, and even BDs discriminate better between lineages. The algorithms for identifying SEs and BDs may cause the differences. So far, the algorithm based on ROSE (Whyte et al., 2013) is widely used for SE identification, while the other algorithms rarely have not been found in previous studies (Whyte et al., 2013; Wei et al., 2016; Perez-Rico et al., 2017). Conversely, various algorithms have been developed for identifying BDs, which may suggest that no one is optimized for all tissues (Benayoun et al., 2014; Chen et al., 2015; Belhocine et al., 2021). Thus, a more optimized algorithm is required for BD identification in future studies. Notably, comparative studies crossing more tissues and more organisms are essential for the determination ability of SEs and BDs.

In summary, our study provides a list of SE and BD candidates for dissecting mechanisms of tissue-specific processes in future studies. It highlights that functionally conserved regulatory elements lack sequence conservation. Furthermore, it demonstrates that SEs are more effective at discriminating tissue identity than BDs, which is different from a previous study.

## Data Availability

The original contributions presented in the study are included in the article/Supplementary Material; further inquiries can be directed to the corresponding author.

## Supplementary Material

Supplementary Table S1 The download information of ChIP-seq and RNA-seq data for pig, human, and mouse tissues.

Supplementary Table S2 Information of SEs for pig, human, and mouse tissues.

Supplementary Table S3 Information of BDs for pig, human, and mouse tissues.

Supplementary Table S4 Analyses of tissue specificity of SEs, TEs, BDs, and NDs.

Supplementary Table S5 The top 20 most significant GO terms for pig, human, and mouse tissues.

Supplementary Table S6 Information of pig functional SEs and BDs in six tissues

Supplementary Figure S1 The intersection of orthologous SEs in six tissues among pig, human, and mouse.

Supplementary Figure S2 The intersection of orthologous BDs in six tissues among pig, human, and mouse.
