# Supplementary figures and images for: A Comparative Analysis of Super-Enhancers and Broad H3K4me3 Domains in Pig, Human, and Mouse Tissues
Source: Front Genet. 2021 Nov 24;12:701049. doi: 10.3389/fgene.2021.701049 (PMC8652260; doi:10.3389/fgene.2021.701049)

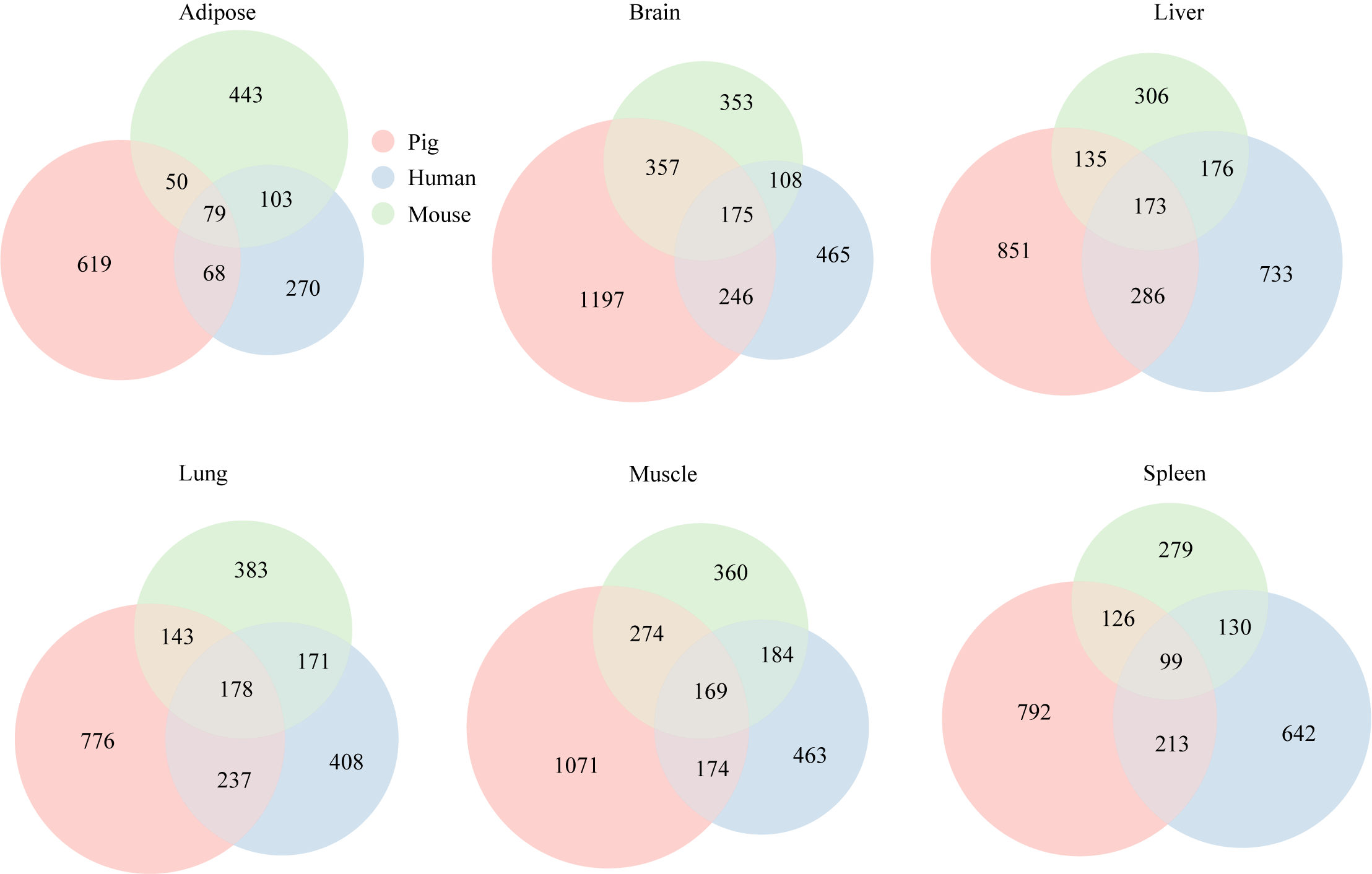

Supplement: Supplementary file 3 [file Image2.TIF]

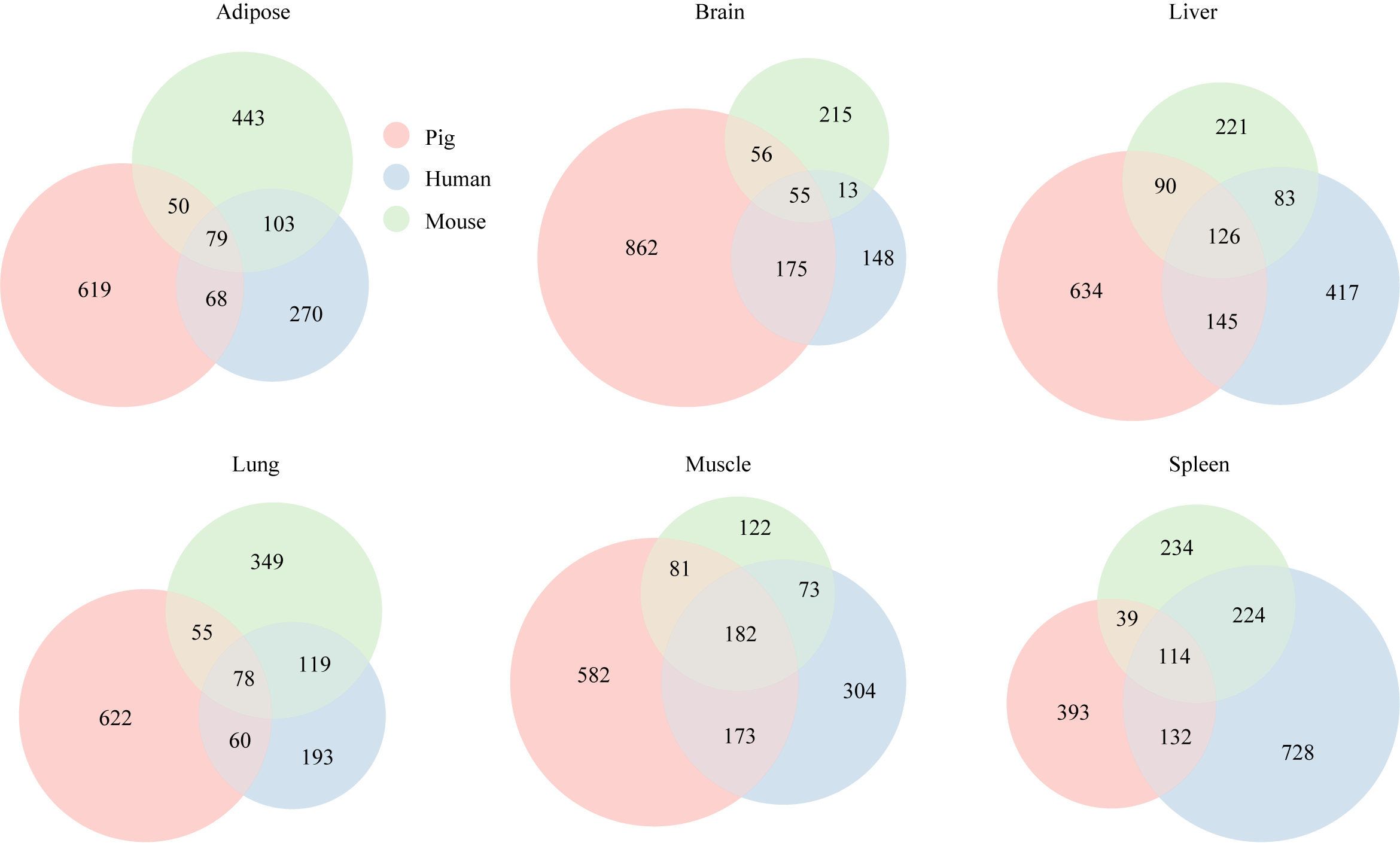

Supplement: Supplementary file 4 [file Image1.TIF]
